# Activation of Basolateral Amygdala to Nucleus Accumbens Projection Neurons Attenuates Chronic Corticosterone-Induced Behavioral Deficits in Male Mice

**DOI:** 10.3389/fnbeh.2021.643272

**Published:** 2021-02-24

**Authors:** Andrew Dieterich, Joseph Floeder, Karina Stech, Jay Lee, Prachi Srivastava, David J. Barker, Benjamin A. Samuels

**Affiliations:** ^1^Neuroscience Graduate Program, Rutgers, The State University of New Jersey, Piscataway, NJ, United States; ^2^Department of Psychology, Behavioral and Systems Neuroscience, Rutgers, The State University of New Jersey, Piscataway, NJ, United States

**Keywords:** basolateral amygdala, nucleus accumbens, instrumental behavior, depression models, chronic stress, reward, motivation

## Abstract

The basolateral amygdala (BLA) is critical for reward behaviors *via* a projection to the nucleus accumbens (NAc). Specifically, BLA-NAc projections are involved in reinforcement learning, reward-seeking, sustained instrumental responding, and risk behaviors. However, it remains unclear whether chronic stress interacts with BLA-NAc projection neurons to result in maladaptive behaviors. Here we take a chemogenetic, projection-specific approach to clarify how NAc-projecting BLA neurons affect avoidance, reward, and feeding behaviors in male mice. Then, we examine whether chemogenetic activation of NAc-projecting BLA neurons attenuates the maladaptive effects of chronic corticosterone (CORT) administration on these behaviors. CORT mimics the behavioral and neural effects of chronic stress exposure. We found a nuanced role of BLA-NAc neurons in mediating reward behaviors. Surprisingly, activation of BLA-NAc projections rescues CORT-induced deficits in the novelty suppressed feeding, a behavior typically associated with avoidance. Activation of BLA-NAc neurons also increases instrumental reward-seeking without affecting free-feeding in chronic CORT mice. Taken together, these data suggest that NAc-projecting BLA neurons are involved in chronic CORT-induced maladaptive reward and motivation behaviors.

## Introduction

Millions of people suffer at least one episode of major depressive disorder (MDD) in their lifetime (Hasin et al., 2005). Chronic stressful life experiences can precipitate the development of mood disorders such as MDD (Hammen, 2005). While numerous antidepressants exist, most actually fail to treat the multitude of diverse and heterogenous symptoms that characterize MDD, especially symptoms related to reward processing and anhedonia (Olfson et al., 2006; Papakostas et al., 2006). Anhedonia is the lack of feelings of pleasure and the positive experience from reward, and is one of the twocore symptoms of MDD, along with depressed mood (Der-Avakian and Markou, 2012; American Psychiatric Association, 2013).

MDD patients display a deficit in reward processing (Pizzagalli et al., 2008; Pechtel et al., 2013). Reward learning and responsiveness to social or monetary rewards are decreased in patients diagnosed with depression (Pizzagalli et al., 2008; Pechtel et al., 2013; Vrieze et al., 2013). This maladaptive reward processing may be due to reduced activation of important brain areas such as the nucleus accumbens (NAc) in depressed patients (Pizzagalli et al., 2009; Dillon et al., 2014). Also, rodents exposed to chronic stress exhibit maladaptive reward and motivation behaviors (Willner et al., 1987; Gourley et al., 2009; Dieterich et al., 2019, 2020). However, the changes in neural circuitry mediating stress effects on maladaptive reward and anhedonia are not fully understood (Russo and Nestler, 2013). A better understanding of this neural circuitry will help elucidate effective treatment strategies that provide targeted therapies to diagnosed patients (Cuthbert and Insel, 2013).

When rodents are exposed to chronic stress, they also exhibit increased avoidance of potentially threatening or aversive environments and contexts (Samuels et al., 2011; Der-Avakian et al., 2015). Historically these changes in avoidance behavior are described as associated with anxiety or depression, even though mood disorders such as anxiety and depression are diagnosed based on self-report of specific symptoms rather than observational data. By contrast, reward and motivated behavioral tests are remarkably similar between rodents and humans. Some rodent tests were even developed to be analogous to already in-use human tests. Therefore, reward and motivation behavioral tests in rodents have direct translational validity that may both improve our understanding of the etiology of mood disorders and better predict treatment outcome (Admon and Pizzagalli, 2015; Der-Avakian et al., 2015). Therefore, understanding how chronic stress causes impairments in neural circuits underlying reward and motivated behaviors in rodents will inform mood disorder research (Cuthbert and Insel, 2013; Der-Avakian and Pizzagalli, 2018).

The basolateral amygdala (BLA) contains heterogeneous populations of glutamatergic principal neurons that mediate aversive and appetitive learning (Schoenbaum et al., 1998; Kim et al., 2016; Beyeler et al., 2018). Subsets of BLA neurons fire selectively in response to either a rewarding sucrose solution or a bitter, aversive quinine solution (Schoenbaum et al., 1998). BLA neurons also fire in response to other rewarding stimuli, aversive foot-shocks, and predictable conditioned stimuli (Cousens and Otto, 1998). These results suggest that BLA neurons mediate conditioned learning and responding to both unconditioned aversive and conditioned appetitive stimuli (Lutas et al., 2019). NAc-projecting glutamatergic BLA neurons are positively reinforcing, promote self-stimulation, and are responsive to conditioned tones predictive of sucrose reward delivery (Amir et al., 2015). Therefore, BLA projections to NAc mediate appetitive conditioning and reward behaviors. However, whether chronic CORT interacts with BLA-NAc projections to mediate maladaptive behaviors remains unknown.

BLA-NAc neurons may bias behavior toward a larger, but riskier reward (Bercovici et al., 2018). Optogenetic inhibition of this projection reduces the selection of smaller, certain rewards and increases the selection of larger, riskier rewards in a probabilistic discounting task (Bercovici et al., 2018). This suggests BLA-NAc neurons signal reward value and promotes high reward responding. In a safe/conflict task where rats lever press for food reward but are shocked during a 5-min “conflict” block. BLA or NAc shell inhibition increases foot-shock, punishes reward-seeking and memory (Piantadosi et al., 2017), without affecting response. BLA neurons likely increase reward-seeking by facilitating NAc activation in response to reward-predictive cues (Stuber et al., 2011; Namburi et al., 2015, 2016).

Chronic CORT administration is a useful paradigm that mimics the effects of other chronic stress paradigms on behavior in males, including maladaptive increases in avoidance. CORT also blunts reward and motivated behaviors (Zhao et al., 2008; David et al., 2009; Gourley et al., 2009, 2012; Dieterich et al., 2019, 2020). Chronic CORT increases latency to eat in the novelty suppressed feeding (NSF; David et al., 2009; Dieterich et al., 2019), a conflict-based feeding task typically associated with avoidance. Chronic CORT administration shifts effortful responding (Dieterich et al., 2020) and also impairs several instrumental reward behaviors, including progressive ratio, outcome devaluation, and probabilistic reversal learning (Gourley et al., 2009, 2012; Dieterich et al., 2019).

To modulate BLA-NAc projections, we used designer receptors exclusively activated by designer drugs (DREADDs) (Armbruster et al., 2007). BLA-NAc chemogenetic control of circuitry was achieved using a dual-virus approach with a retrograde-transporting Cre-recombinase virus infused into the NAc, and a Cre-dependent DREADD virus infused into the BLA. We reasoned that chemogenetic manipulation of BLA-NAc neurons would have bidirectional effects on reward behaviors without impacting avoidance. Furthermore, we hypothesized that modulating BLA projections to the NAc could reverse the effects of chronic CORT on motivated behaviors.

## Materials and Methods

### Animals

One-hundred and eighteen adult male C57BL/6J mice from Jackson Labs (Bar Harbor, ME, USA) were maintained on a 12 light:12 dark schedule. All experiments followed NIH laboratory animal care guidelines and were approved by the Rutgers University Institutional Animal Care and Use Committee (IUCAC). Mice were randomly divided into a BLA-NAc cohort and two separate CORT administration cohorts. In the BLA-NAc cohort, mice were assigned to mCherry (*n* = 10), Gq-DREADD (*n* = 10), and Gi-DREADD (*n* = 10) groups. In the two CORT administration experiments, mice in each cohort were assigned to Vehicle mCherry (*n* = 10), Vehicle Gq-DREADD (*n* = 10), CORT mCherry (*n* = 10), and CORT Gq-DREADD (*n* = 10) groups. All behavioral experiments were conducted between 9:00 AM and 3:00 PM daily, which was during the light phase.

### Stereotaxic Surgeries and Viral Infusions

Mice were anesthetized with 1–1.5% isoflurane and placed in a stereotax (Kopf Instruments, Tujunga, CA, USA). A vertical incision was made at the midline of the skull and all connective tissue covering the skull was removed. Alternating betadine and 70% ethanol washes were used to disinfect and clean the top of the skull. Then, 8-week-old mice were bilaterally infused into the NAc with 300 nl of the retrograde pENN.AAVrg.hSyn.HI.eGFP-Cre.WPRE.SV40 virus (AddGene Plasmid # 105540, Watertown, MA, USA), virus titer: (≥7 × 10^12^ vg/ml). For NAc infusions, the coordinates were: 1.30 mm anterior to Bregma, 1.00 mm lateral to the midline, and 4.60 mm ventral to skull surface (Supplementary Figure 4B). Next, mice were bilaterally infused with 300 nl of a Cre-sensitive Gq-DREADD [pAAV-hSyn-DIO-hM3D(Gq)-mCherry, Addgene Plasmid # 44361], virus titer: (≥7 × 10^13^ vg/ml), Gi-DREADD [pAAV-hSyn-DIO-hM4D(Gi)-mCherry, Addgene Plasmid # 443632; virus titer ≥1 × 10^13^ vg/ml], or mCherry virus (pAAV-hSyn-mCherry, Addgene Plasmid # 114472), virus titer: (≥1 × 10^13^ vg/ml), into the BLA at the following coordinates: 1.40 mm posterior to Bregma, 3.00 mm lateral to midline, and 4.90 mm ventral to the skull surface (Supplementary Figure 4A; Krashes et al., 2011). These virus titers and stereotaxic coordinates remained the same for all cohorts and in all groups. Mice were given 4 weeks to recover and ensure viral expression before habituation and training in the operant chambers and other behavioral testing.

### Corticosterone Administration

In the two chronic CORT administration cohorts, Vehicle (*n* = 20) and CORT-administered (*n* = 20) mice began Vehicle or CORT administration at the time of viral infusions. After 4 weeks of CORT administration, mice began behavior testing, which lasted an additional 3 weeks. CORT-administered mice received CORT (35 μg/ml; Sigma–Aldrich, St. Louis, MO, USA) and beta-cyclodextrin (4.5 mg/ml) dissolved in the drinking water. This paradigm produces a CORT dose of approximately 9.5 mg/kg/day (Dieterich et al., 2019). Vehicle-administered mice only received beta-cyclodextrin in the drinking water but no CORT. Beta-cyclodextrin is a palatable but non-caloric sugar often dissolved in the drinking water along with CORT (David et al., 2009). One cohort was trained to lever press and was tested in FR30 and progressive ratio tests, followed by open field and NSF. The second cohort was tested in free-feeding and instrumental reward-seeking tasks. In the two cohorts, body weights (Supplementary Figures 3D,G) and liquid consumed (Supplementary Figures 3E, 4H) and mean daily CORT dose (Supplementary Figures 3F,I) were recorded across 4 weeks of CORT administration and then 3 weeks of behavior testing.

### Clozapine N-Oxide (CNO)

CNO (Sigma–Aldrich, St. Louis, MO, USA) was dissolved in 1 ml of DMSO and adjusted to a final concentration of 0.5 mg/ml in 7.5% v/v DMSO diluted in ddH_2_O. On all behavior testing days, mice received an intra-peritoneum (i.p.) injection with 2 mg/kg CNO, 30 min before testing (Jendryka et al., 2019). A group of BLA mCherry saline pretreatment control mice not injected with CNO (*n* = 8) in the BLA-NAc experiments was used to confirm no effects of CNO on behavior.

### Instrumental Chambers

Mice were trained to lever press and were then tested in standard mouse instrumental chambers (Med Associates, Fairfax, VT, USA) housed in sound-attenuating cubicles, in a separate behavioral testing room. The chambers were coupled to power control and interface connected to a computer running Med-PC IV software (Med Associates, Fairfax, VT, USA). The instrumental chambers contained two retractable response levers along one wall, and 220 mg food pellet hoppers attached by *Y*-tubing to a single reward port in-between the two levers delivering food pellets for consumption (Bio-Serv, Flemington, NJ, USA).

### Instrumental Conditioning

After 4 weeks of surgery recovery and vehicle or CORT administration, mice were food-restricted to 90% of their free-feeding body weights. Mice were initially trained on a continuous reinforcement schedule where every lever press was reinforced with the delivery of a single reinforcer pellet. After reaching the acquisition of the instrumental response (15 or more lever presses in a 20-min session), mice were trained for two sessions on an FR10 operant task where every tenth lever press was reinforced with a reinforcer pellet. The BLA-NAc cohort completed a 1-h progressive ratio (PROG) test to examine the effect of activating or inhibiting this circuit on motivation to work for the reinforcer. The PROG reinforcement schedule increased exponentially (5, 10, 20, 30, 50, et cetera), a commonly used PROG schedule (Celentano et al., 2009; De Luca and Badiani, 2011; Cui et al., 2012; Davis et al., 2012). Breakpoint was determined as the point when the mouse stopped lever pressing and was recorded in addition to total active lever presses. In the CORT administration cohort, mice were trained similarly on an FR10 schedule and then tested in a 30-min FR30 session. After all operant conditioning training and testing sessions, mice were returned to the colony room and given standard lab chow at least 1-h later.

### Open Field (OFT)

The OFT test was conducted in standard Plexiglas open-field chambers (43 × 43 cm) in a separate behavioral testing room. Motor Monitor software (Kinder Scientific, Poway, CA, USA) was used to record mouse activity in a 15-min test. Infrared photo beams surrounding the chamber detected mouse movement. Locomotor activity, center time, and center distance were recorded.

### Novelty Suppressed Feeding (NSF)

Mice were food-deprived for 18 h, and then placed in the corner of a novel brightly lit (1,500 lux) apparatus filled with corncob bedding. A single food pellet was placed in the center of the arena directly under the light. The NSF test lasted up to 6 min, and latency to take a bite of the pellet was recorded. Mice that timed-out, and did not eat, were assigned a latency of 360 s. After 6 min, mice were transferred to their home cages and given *ad libitum* access to a single food pellet for 5 min. Latency to eat and amount of the pellet consumed in the home cage were recorded, as control behaviors for the NSF test.

### Sucrose Preference Test (SPT)

Mice in the BLA-NAc cohort were habituated to a 1% sucrose solution in their home cage for 3 days. Then, mice were habituated to a fresh cage containing two side-by-side water bottles for a 2-h habituation session. The following day, mice were injected with 2 mg/kg CNO 30-min before testing and placed in a fresh cage containing one water bottle and one bottle containing 1% sucrose. Consumption of both water and sucrose bottles was measured by weighing the bottles before and after testing. The chronic CORT cohort was not tested in SPT, as both Vehicle and CORT water bottles contained the artificial sweetener beta-cyclodextrin.

### Instrumental Reward-Seeking Task

In one of the chronic CORT administration cohorts, mice were habituated for 30 min to an instrumental chamber. Mice were then food-deprived overnight for 18 h and injected with 2 mg/kg CNO (i.p.) 30 min before a 30-min instrumental nose-poke reward-seeking task. Nose pokes into the reward port for a food pellet triggered a subsequent pellet delivered after a 10-s delay. The sessions began with a reward pellet in the reward port. The number of reinforcers earned was recorded in the 30-min session.

### Feeding Behavior

In the chronic CORT administration cohort tested in reward-seeking, mice were also tested in a 2-h free-feeding test to examine the effect of chronic CORT and chemogenetic BLA-NAc neuron activation on feeding behavior. Mice were first habituated to a test cage to measure free-feeding behavior. All mice were then food-deprived for 18 h, injected with 2 mg/kg CNO (i.p.), and after 30 min were placed in individual cages containing a single large food pellet weighing 3–4 g. The weight of food consumed was measured after 2 h.

### Transcardial Perfusions

After completing behavior testing, all mice were transcardially perfused with 4% PFA and whole brains were extracted, approximately 90 min after 2 mg/kg CNO injection. Whole brains were sectioned (40 μm sections) on a cryostat (Leica Biosystems, Buffalo Grove, IL, USA), washed with 1× PBS, stained with 4^′^,6-Diamidino-2-Phenylindole, Dihydrochloride (DAPI; Thermo Fisher Scientific, Waltham, MA, USA) to allow visualization of nucleic acid within cell nuclei, washed with 1× PBS, cover-slipped with Prolong Diamond Antifade Mountant (Thermo Fisher Scientific, Waltham, MA, USA), and imaged on a fluorescence microscope (EVOS Auto 2.0, Thermo Fisher Scientific, Waltham, MA, USA) to determine the accuracy of viral infusions. The retrograde-Cre virus fluoresced green *via* an attached GFP conjugate. The Gi- and Gq-DREADD viruses fluoresced red *via* conjugated mCherry tag.

### Statistical Analyses

Statistical analyses were conducted using GraphPad Prism (GraphPad Prism Software, La Jolla, CA, USA). One-way ANOVAs with planned multiple comparisons were used to determine significance between each DREADD group (Gq- or Gi-) and the control (mCherry) group in each behavioral test for the BLA-NAc cohort. Two-way ANOVAs with DREADD group and CORT treatment as between-subjects factors were used to compare Vehicle and CORT-administered mice expressing either mCherry or Gq-DREADD in the BLA-NAc circuit. Planned multiple comparisons were made to determine significance between groups. Data are presented as mean ± SEM, and significance is considered as *p* < 0.05.

## Results

### BLA-NAc Effects on Behavior in the Absence of Chronic CORT

**Figure 1:**
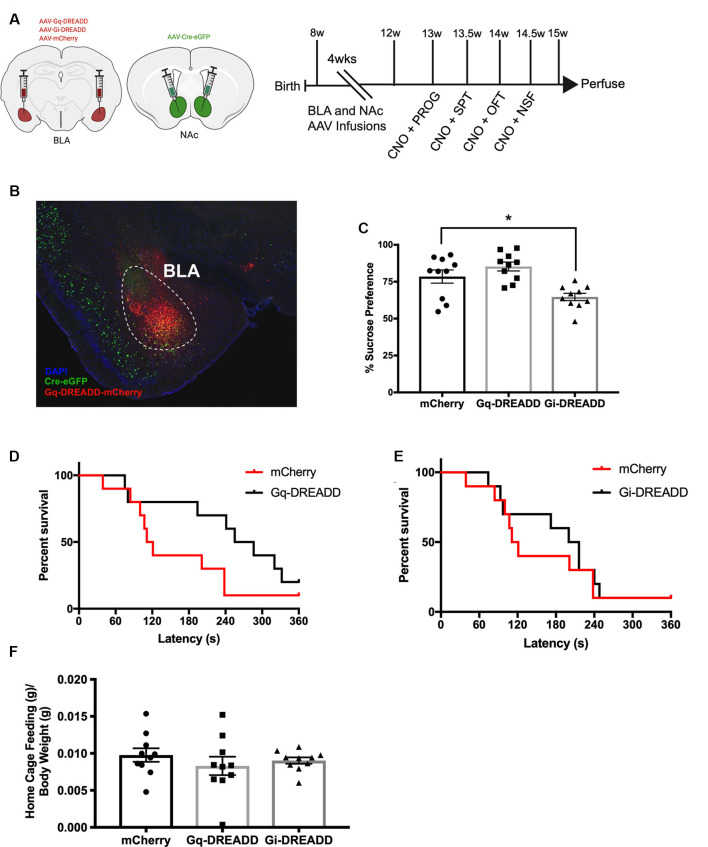
Inactivating basolateral amygdala-nucleus accumbens (BLA-NAc) neurons reduces sucrose preference.** (A)** Timeline of BLA-NAc experiment. Schematic indicates BLA and NAc viral infusions (AAV-retrograde-eGFP-Cre: green; AAV-DIO-DREADD: red). **(B)** Merged image taken at 4× magnification of BLA-NAc neurons expressing AAV-Cre-eGFP and AAV-DREADD-mCherry. **(C)** BLA-NAc inhibition reduces preference for sucrose in sucrose preference test (SPT). **(D)** Activating BLA-NAc neurons does not affect novelty suppressed feeding (NSF) latency. **(E)** Inactivating BLA-NAc neurons does not affect NSF latency. **(F)** Weight of a food pellet eaten in the 5-min home cage measure of novelty suppressed feeding (NSF) compared to body weight is similar across groups. Bars are mean ± SEM. *p < 0.05.

We began by assessing whether BLA-NAc projection neurons modulate avoidance and reward behaviors. Eight-week-old C57BL/6J male mice were infused with Cre-dependent Gq-DREADD, Gi-DREADD, or mCherry viruses in BLA and retrograde Cre virus in NAc (Figures 1A,B; Supplementary Figure 4) and starting 4 weeks later were trained and then tested in a progressive ratio (PROG), sucrose preference (SPT), OFT, and NSF (Figure 1A). For PROG, separate one-way ANOVAs revealed no effect of chemogenetic modulation of BLA-NAc projection neurons on number of active lever presses (*F*_(2,27)_ = 0.23, *p* = 0.7976; Supplementary Figure 1A), on PROG breakpoint reached (*F*_(2,27)_ = 0.90, *p* = 0.417; Supplementary Figure 1B), or on reinforcers earned (g) divided by body weight (g; *F*_(2,27)_ = 1.50, *p* = 0.2323; Supplementary Figure 1C). For SPT, a one-way ANOVA revealed an effect of BLA-NAc projection neurons in preference for a 1% sucrose solution (*F*_(2,27)_ = 9.40, *p* = 0.0008; Figure 1C). Compared to mCherry control, inhibiting BLA-NAc projection neurons (Gi-DREADD) reduced preference for sucrose (*p* = 0.0163). However, compared to mCherry control, activating the BLA-NAc circuit (Gq-DREADD) did not significantly alter sucrose preference (*p* = 0.3432). For sucrose consumption in SPT (ml) compared to mouse body weight (g) in the BLA-NAc cohort, consumption per gram body weight (g) was similar between groups (*F*_(2,27)_ = 1.60, *p* = 0.2277; Supplementary Figure 1D).

For OFT, a one-way ANOVA revealed no effect of BLA-NAc projection neurons on distance traveled in the OFT (*F*_(2,27)_ = 3.3, *p* = 0.0541; Supplementary Figure 1F). A one-way ANOVA also revealed no effect of BLA-NAc projection neurons on time in the center of the OFT arena (*F*_(2,27)_ = 0.16, *p* = 0.8563; Supplementary Figure 1G). For NSF, separate Kaplan Meier Survival Analyses were conducted to compare the effect of activating or inhibiting BLA-NAc neurons on latency to eat in the NSF. Compared to mCherry control, activating (Gq-DREADD) BLA-NAc neurons did not affect latency to eat (Gq: $X_{\text{(1)}}^{\text{2}}$ = 2.6, *p* = 0.1059; (Figure 1D). Inactivation of BLA-NAc neurons did not affect NSF latency to eat (Gi: $X_{\text{(1)}}^{\text{2}}$ = 0.29, *p* = 0.5891; Figure 1E). For NSF home cage pellet consumption (g) divided by body weight (g), a one-way ANOVA was not significant (*F*_(2,27)_ = 0.63, *p* = 0.5420; Figure 1F), indicating all DREADD groups consume food similarly in the NSF home cage. When NSF latency was divided by home cage pellet consumption and expressed as a ratio, BLA-NAc activation did increase the ratio compared to mCherry control (Supplementary Figure 1E). To confirm that CNO administration did not have effects in the absence of DREADDs, we compared the BLA-NAc mCherry group to an identical BLA-NAc mCherry group (*n* = 8) injected with an equal volume of saline 30 min before each behavior (Supplementary Figure 2). We did not see significant effects of CNO administration relative to saline in PROG, SPT, OFT, NSF, or home cage feeding. Taken together, inhibiting BLA-NAc projection neurons reduced preference for sucrose, without affecting instrumental reward responding, locomotion, or avoidance behavior.

### BLA-NAc Effects on Reward, NSF, and Avoidance in the Presence of Chronic CORT

**Figure 2:**
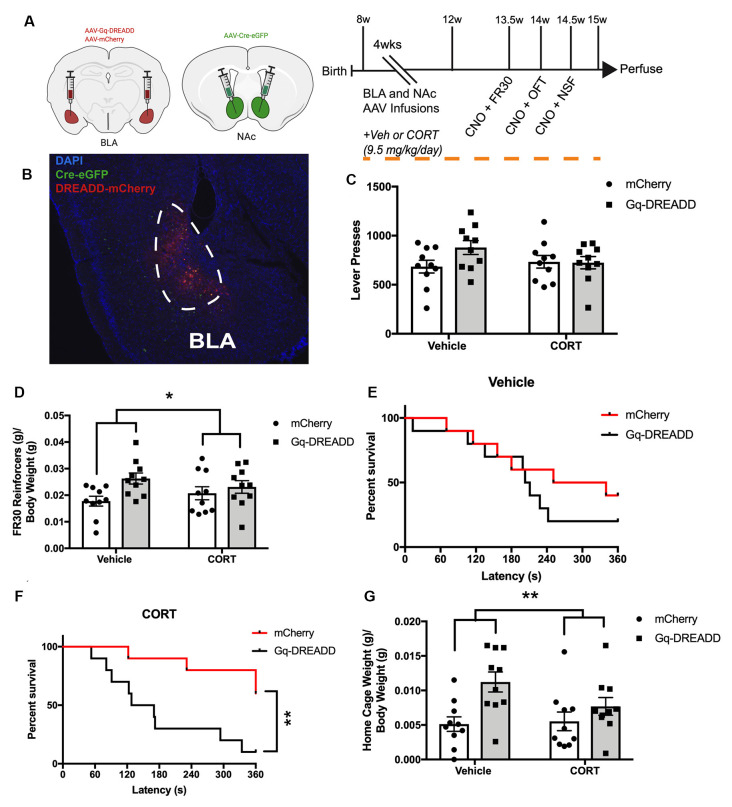
Activating BLA-NAc neurons rescues CORT-induced NSF latency. **(A)** Timeline of BLA-NAc CORT administration experiment and schematics indicating AAV viral infusions into BLA or NAc (AAV-retrograde-eGFP-Cre: green; AAV-DIO-Gq-DREADD: red). **(B)** Merged image at 4× magnification of BLA-NAc neurons expressing AAV-Cre-eGFP and AAV-DREADD-mCherry. **(C)** In an FR30 test, BLA-NAc neurons do not affect lever pressing in Vehicle or chronic CORT mice. **(D)** FR30 reinforcers earned (g) per gram body weight (g) is increased by BLA-NAc activation. **(E)** BLA-NAc activation does not affect NSF latency in Vehicle mice. **(F)** BLA-NAc activation rescues chronic CORT-induced increase in NSF latency. **(G)** Home cage weight (g) of food pellet eaten compared to body weight (g), in a 5-min test in the BLA-NAc CORT cohort is increased by activating BLA-NAc neurons but not affected by chronic CORT. Bars are mean ± SEM. *p < 0.05; **p < 0.01.

We next wanted to explore whether stimulating BLA-NAc projection neurons could reverse the effects of chronic CORT on maladaptive behaviors (Stuber et al., 2011; Namburi et al., 2015, 2016). Thus, we activated BLA-NAc projection neurons with Gq-DREADDs in mice administered chronic CORT (Figure 2A). Eight-week-old adult male C57BL/6J mice were infused with AAV Gq-DREADD or mCherry in the BLA and retrograde AAV Cre in the NAc, which leads to specific DREADD expression in BLA-NAc projection neurons (Figure 2B). All mice were administered CORT or Vehicle for the 4 weeks following viral infusions and then tested in fixed ratio 30 (FR30), OFT, and NSF. For the FR30 test, a two-way ANOVA with BLA-NAc activation and CORT administration as between-subjects factors revealed no main effect of BLA-NAc neurons on lever presses (*F*_(1,36)_ = 2.0, *p* = 0.167), no main effect of CORT administration (*F*_(1,36)_ = 0.64, *p* = 0.430), and no interaction (*F*_(1,36)_ = 2.4, *p* = 0.129; Figure 2C). For reinforcers consumed in the FR30 task compared to mouse body weight (g/g), a two-way ANOVA revealed a main effect of BLA-NAc activation (*F*_(1,36)_ = 6.10, *p* = 0.019), no main effect of CORT administration (*F*_(1,36)_ = 0.0019, *p* = 0.966), and no interaction (*F*_(1,36)_ = 1.90, *p* = 0.174; Figure 2D). Thus, when FR30 responding was examined in comparison to animal weight, BLA-NAc activation increased reinforcers earned, while chronic CORT did not affect reinforcers obtained, while lever presses were not influenced by BLA-NAc activation.

For OFT, a two-way ANOVA with BLA-NAc activation and CORT-administration as between-subjects factors revealed no main effect of CORT administration on locomotion (*F*_(1,36)_ = 0.38, *p* = 0.542) no main effect of BLA-NAc activation (*F*_(1,36)_ = 0.43, *p* = 0.515, and no interaction (*F*_(1,36)_ = 0.00019, *p* = 0.965; Supplementary Figure 3A). For time in the center of the OFT, a measure of anxiety behavior, a two-way ANOVA revealed no main effect of CORT (*F*_(1,36)_ = 0.86, *p* = 0.361), no main effect of BLA-NAc neurons (*F*_(1,36)_ = 4.0, *p* = 0.054), and no interaction (*F*_(1,36)_ = 0.032, *p* = 0.860; Supplementary Figure 3B), though BLA-NAc activation trended to increase OFT time in the center.

In NSF, separate Kaplan Meier Survival Analyses were conducted for Vehicle and CORT-administered mice to determine the effect of BLA-NAc activation on latency to eat in the NSF. Vehicle-administered BLA-NAc mCherry and BLA-NAc Gq-DREADD mice did not differ in latency to eat (Log-rank Mantel-Cox test; $X_{\text{(1)}}^{\text{2}}$ = 1.0, *p* = 0.3180; Figure 2E). However, in CORT-administered mice, BLA-NAc mCherry and BLA-NAc Gq-DREADD mice differed significantly in latency to eat in the NSF (Log-rank Mantel-Cox test; $X_{\text{(1)}}^{\text{2}}$ = 8.50, *p* = 0.0036; Figure 2F). Thus, BLA-NAc activation reduces NSF latency in CORT-administered mice without impacting latency in Vehicle mice. For NSF home cage pellet consumption compared to body weight (g/g), a two-way ANOVA revealed a main effect of BLA-NAc activation (*F*_(1,36)_ = 10.0, *p* = 0.003), no main effect of CORT administration (*F*_(1,36)_ = 1.50, *p* = 0.234), and no interaction (*F*_(1,36)_ = 2.30, *p* = 0.138; Figure 2G). Thus, when NSF home cage feeding was examined by body weight, BLA-NAc activation increased pellet consumption, while chronic CORT did not affect the NSF home cage control feeding behavior. NSF ratio was calculated similar as above and there were no group differences, though chronic CORT had a small trend to increase the ratio (*p* = 0.080; Supplementary Figure 3). Thus, activation of BLA-NAc projection neurons reverses the effects of chronic CORT in NSF and has nuanced effects on home cage feeding.

### Chronic CORT-Administered BLA-NAc Reward Seeking and Feeding

**Figure 3:**
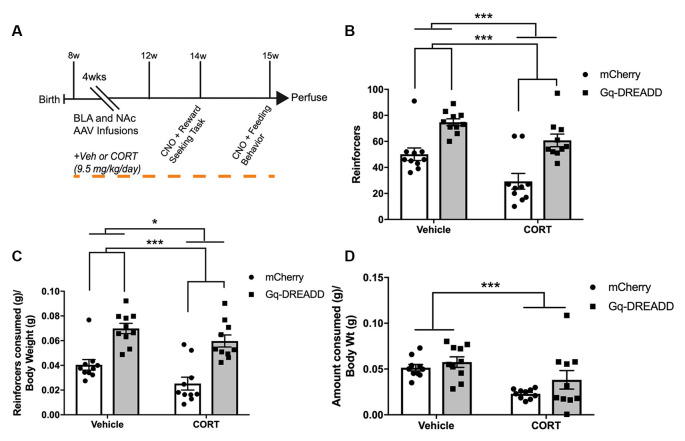
Activating BLA-NAc neurons increases chronic CORT deficit in reward-seeking.** (A)** Timeline of BLA-NAc CORT administration experiment. **(B)** In a 30-min instrumental nose-poke task, chronic CORT reduces the number of reinforcers consumed while BLA-NAc activation increases reinforcers eaten in both Vehicle and CORT mice. **(C)** Nose-poke task reinforcers consumed (g) normalized to body weight (g). **(D)** In a 2-h free-feeding test, chronic CORT reduces the amount of lab chow consumed (grams) per gram body weight. Chemogenetic activation of BLA-NAc neurons does not affect feeding behavior. Bars are mean ± SEM. *p <0.05; ***p < 0.0001.

We next wanted to understand how CORT administration and activation of BLA-NAc projection neurons affects reward-seeking and free feeding. Eight-week-old adult male C57BL/6J mice were infused with AAV Gq-DREADD or mCherry into the BLA, and retrograde AAV Cre into the NAc. All mice were administered CORT or Vehicle for 4 weeks following viral infusions and then tested in instrumental reward-seeking and free feeding tasks (Figure 3A). In an instrumental nose-poke reward-seeking task, where each nose poke triggered delivery of a subsequent food reward after 10 s, a two-way ANOVA with CORT administration and BLA-NAc activation as between-subjects factors revealed a significant main effect of BLA-NAc activation (*F*_(1,36)_ = 34.87, *p* < 0.001), a main effect of CORT (*F*_(1,36)_ = 13.34, *p* = 0.0008), but no interaction (*F*_(1,36)_ = 0.5275, *p* = 0.4723; Figure 3B) in the number of reinforcers earned. Thus, CORT administration significantly reduced the number of reinforcers earned, and activation of BLA-NAc projection neurons increased reinforcers earned in a 30-min reinforced nose-poke operant task. To confirm these differences were not due to animal body weight affecting behavior, when reinforcers consumed (g) were compared to body weight (g), a two-way ANOVA revealed a significant main effect of BLA-NAc activation (*F*_(1,36)_ = 7.356, *p* = 0.0102), a main effect of CORT (*F*_(1,36)_ = 47.0, *p* < 0.0001), but no interaction (*F*_(1,36)_ = 0.2994, *p* = 0.5876) on reinforcer consumption normalized to body weight (Figure 3C).

In the free-feeding task, a two-way ANOVA with CORT administration and BLA-NAc activation as between-subjects factors revealed a main effect of CORT (*F*_(1,36)_ = 15.24, *p* = 0.0004), no main effect of BLA-NAc activation (*F*_(1,36)_ = 3.039, *p* = 0.0898), and no interaction (*F*_(1,36)_ = 0.5512, *p* = 0.4626) on amount of lab chow consumed per gram body weight (Figure 3D). Thus, chronic CORT administration reduces the amount of food consumed in a 2-h free-feeding test after 18 h of food deprivation. Taken together, these data suggest that BLA-NAc activation affects reward-seeking but not free feeding.

## Discussion

Here we show a nuanced role for how BLA-NAC projection neurons mediate reward, motivation, and avoidance behaviors. In the absence of CORT, chemogenetic targeting of BLA-NAc projections affected reward behavior without directly impacting avoidance. Specifically, inhibiting BLA-NAc projections reduced sucrose preference. A reduction in preference for sucrose is historically described as increased anhedonic behavior (Willner et al., 1987). By contrast, in mice subjected to chronic CORT administration, Gq-DREADD-mediated activation of BLA-NAc neurons reversed the CORT-induced increase in NSF latency, a task typically associated with avoidance. BLA-NAc activation also enhanced instrumental reward-seeking in both Vehicle and CORT mice without affecting free-feeding behavior. Taken together, these data demonstrate a nuanced role for BLA projections to the NAc in mediating motivated reward behaviors in the absence or presence of chronic CORT.

### Distinct BLA Projections in Avoidance and Reward Behaviors

Avoidance and motivated behaviors are thought to be mediated by separate glutamatergic BLA projections to multiple regions, including the vHIP and NAc, respectively (Schoenbaum et al., 1998). Our data show nuanced effects of BLA-NAc neurons in mediating reward behavior (Stuber et al., 2011; Britt et al., 2012; Namburi et al., 2015). These differences are likely caused entirely by methodological differences, as we used a dual virus chemogenetic approach while others used single virus optogenetic approaches with localized photoactivation or inhibition. BLA-NAc neurons affect reinforcement and natural reward seeking using optogenetic targeting of this circuit (Stuber et al., 2011). In an operant self-stimulation task, mice expressing ChR 2 but not eYFP in these neurons rapidly learn to nose poke for further BLA-NAc stimulation (Stuber et al., 2011). BLA-NAc neurons were similarly found self-reinforcing in an operant nose poke task as well as in a place preference task (Britt et al., 2012). Conversely, NpHR-mediated optogenetic inhibition of this circuit reduces the lick rate for sucrose delivery (Stuber et al., 2011). BLA-NAc neurons are also self-reinforcing in an optogenetic self-stimulation paradigm (Namburi et al., 2015). Our results align with these findings, particularly in the CORT administration experiments, as BLA-NAc activation increased the number of reinforcers consumed in the instrumental nose-poke reward-seeking task. BLA-NAc effects likely depend on dopamine D1-type receptor signaling (Stuber et al., 2011). BLA-NAc activation may drive these effects on reward behavior by increasing dopaminergic activation in the NAc *via* a D1-receptor mechanism. BLA-NAc neurons also undergo synaptic plasticity during reward learning, indicating a broad and central role of this projection in mediating reward behavior (Namburi et al., 2015). Our results provide some details about how chronic stress affects reward behaviors and how NAc-projecting BLA neurons have differential effects on behavior in the presence or absence of chronic stress. Similar to previous reports (David et al., 2009; Dieterich et al., 2019), CORT administration produced a robust increase in NSF latency and a blunting of instrumental reward seeking. These maladaptive CORT-induced deficits were attenuated by BLA-NAc activation. Therefore, NAc-projecting BLA neurons are a potential therapeutic target for the treatment of maladaptive chronic stress-induced changes in behavior and reward circuitry.

Importantly, different subpopulations of BLA neurons mediate motivational states and have distinct contributions to behavior (Shen et al., 2019). At least two populations of BLA neurons [cholecystokinin (CCK+) and non-CCK] project to NAc. CCK+ projections synapse on dopamine receptor 2 (DR2) medium spiny neurons (MSNs) in NAc, while non-CCK projections synapse primarily on dopamine receptor 1 (DR1) MSNs. Optogenetic activation of CCK+ BLA-NAc projection neurons is aversive, while activation of non-CCK projections produces strongly reinforced behavior and optical self-stimulation (Shen et al., 2019). Interestingly, exposure to chronic social defeat stress specifically activates CCK+ BLA-D2 NAc projections in susceptible mice (Shen et al., 2019). Future studies are necessary to address whether CORT administration has similar effects, and which subpopulation of BLA-NAc projection neurons is responsible for reversing CORT-induced effects.

### NSF Provides a Readout of Both Avoidance and Motivated Reward Neural Circuitry

We did not anticipate that BLA-NAc activation would reverse CORT-induced effects on NSF because BLA-vHIP projections are reportedly more important for innate avoidance behaviors than BLA-NAc (Felix-Ortiz et al., 2013; Felix-Ortiz and Tye, 2014; Beyeler et al., 2016). However, BLA-NAc projections are critical for two-way avoidance in which rodents avoid an aversive unconditioned stimulus in a divided chamber task (Moscarello and Maren, 2018). BLA-NAc projections are also critical for the acquisition of fear extinction and may renew reward-seeking (Correia et al., 2016; Moscarello and Maren, 2018). NSF is an approach-avoidance task where a food-deprived mouse must enter a brightly lit and innately aversive center of an arena to access a food pellet (Samuels and Hen, 2011). Chronic stress increases latency to eat (David et al., 2009; Dieterich et al., 2019; Yohn et al., 2019a, b) and historically NSF is associated with anxiety behavior because anxiolytics and antidepressants decrease latency to eat (David et al., 2009). However, NSF forces the subject to balance risk with food reward in a highly motivated, food-deprived state. There is a conflict between avoidance of a novel, aversive environment and potential risk associated with entering the center to consume the food, and the rewarding aspect of feeding given a state of food deprivation (Samuels and Hen, 2011). Thus, NSF could arguably also be classified as a reward or motivation task. We found that activation of NAc-projecting BLA neurons, which we hypothesized would specifically affect motivation and reward behaviors, reversed the maladaptive chronic CORT-induced increase in NSF latency to eat. Therefore, NSF may be a readout of both innate avoidance and reward neural circuitry. An alternative explanation is that this effect in NSF may be due to BLA-NAc activity-induced increases in feeding behavior. Activation of BLA-NAc neurons increased food consumption in the home cage immediately following NSF, so we cannot rule this possibility out. Qualitatively the main effect of BLA-NAc activation in the NSF home cage appears to be mostly due to increased consumption in the Vehicle mice, while mCherry and Gq-DREADD CORT mice appear to consume similarly. However, since we did not observe an interaction, we did not perform* post hoc* multiple comparisons. We also did not see a similar effect on home cage feeding of Gq-mediated activation of BLA-NAC projection neurons in vehicle-treated mice from the initial cohort (Figure 1E) or an effect of Gq-mediated activation of BLA-NAC projection neurons on free feeding (Figure 3C). Thus, we saw a nuanced effect whereby BLA-NAc activation appears to reverse the maladaptive effects of chronic CORT on NSF.

### Chronic CORT and Feeding Behavior

Chronic stress may affect feeding behavior. Mice on a high-fat diet display altered corticosterone levels in their plasma and attenuated effects of chronic stress on avoidance behavior (Finger et al., 2011). Acute treatment with 3, 10, and 30 mg/kg corticosterone dose-dependently increases feeding across 4–8 h (Ge et al., 2017). However, others find that acute stress caused by restraint or forced swim reduces feeding in rats and mice (Compan et al., 2004; Calvez et al., 2011). Five days of corticosterone injection into chickens and frogs increases overall food consumption (Nasir et al., 1999; Crespi et al., 2004) and consumption of more palatable food (Pecoraro et al., 2004). 15 or 30 mg of corticosterone in a cholesterol pellet increased food intake in rats (Arvaniti et al., 1998). Corticosterone may initially reduce body weight due to an increase in leptin, a hormone important for energy balance and inhibition of feeding *via* effects in the arcuate of the hypothalamus (Morash et al., 1999; Gemmill et al., 2003). With food deprivation, leptin levels decrease, contributing to an increase in hunger and feeding behavior (Morash et al., 1999). Corticotropin-releasing factor 1 (CRF-1) infusions into the BLA decrease feeding behavior in a 30-min free-feeding task, mimicking the suppression of feeding caused by predator exposure (Jochman et al., 2005). The NAc has a broad role in mediating feeding behavior, with the shell region particularly implicated in stimulating feeding (Maldonado-Irizarry et al., 1995). Taken together, these findings suggest an interaction between feeding and CORT and a potential role of BLA-NAc neurons in mediating this feeding behavior. Here we found that activation of BLA-NAc neurons in CORT-treated mice increased consumption of food pellets in an instrumental reward-seeking task, without affecting overall free-feeding behavior in a 2-h feeding control behavior. Chronic CORT administration reduced both the number of reinforcer pellets consumed in the reward-seeking task as well as total lab chow consumed in the 2-h free-feeding test. However, since CORT has direct effects on feeding, future studies need to use other chronic stress paradigms to better establish the link between stress and feeding.

The chronic CORT administration paradigm was originally developed based on the idea that HPA axis activation is observed in depression patients and can be corrected with chronic antidepressant treatments (Yehuda et al., 1996; Holsboer, 2001; Putman et al., 2010). In rodents, chronic CORT administration produces a sustained increase in plasma CORT levels (Wong et al., 2000; Johnson et al., 2006; Zhao et al., 2008; David et al., 2009; Gourley et al., 2009). Several distinct chronic stress paradigms are widely used in rodents, including chronic unpredictable/variable mild stress, chronic social defeat stress, chronic social instability, and chronic restraint stress (Galea et al., 1997; Golden et al., 2011; Samuels et al., 2011; Yohn et al., 2019a). These paradigms, and chronic CORT administration, all yield maladaptive behaviors that are suitable for assessing the pharmacology and neural circuitry of the antidepressant response (Samuels et al., 2011; Yohn et al., 2020). Chronic CORT also mimics the effects of chronic social defeat stress on intracellular Activin signaling in the dentate gyrus (Gergues et al., 2021). However, one important limitation is that the CORT paradigm is not effective in females (Mekiri et al., 2017; Yohn et al., 2019a). Future studies in both sexes are necessary to assess whether other chronic stress paradigms have similar effects as CORT in males on BLA-NAC projections and behavior.

Another limitation of chronic CORT administration is that we were unable to expand on the sucrose preference effect observed in BLA-NAc mice expressing Gi-DREADD (Figure 1), as chronic CORT is administered through drinking water with the artificial sweetener beta-cyclodextrin to promote intake (David et al., 2009). Thus, interpreting SPT data in these mice would be confounded. Others have used a slightly altered version of the CORT paradigm and found no effect on hedonic responding in SPT (Berger et al., 2019). Given this limitation, we instead used an instrumental reinforcer pellet nose-poke task to test reward-seeking behavior in mice subjected to chronic CORT administration and found a clear effect of activating BLA-NAc neurons in rescuing the CORT-induced reduction in the number of reinforcers consumed.

A potential weakness of the BLA-NAc CORT experiment is that OFT time in the center trended to be different between groups (Supplementary Figure 3; *p* = 0.054). Secondly, in the BLA-NAc cohort, both Gq-DREADD and Gi-DREADD mice appeared to have slightly reduced locomotion (Supplementary Figure 1; *p* = 0.054), suggesting CNO administration and subsequent BLA-NAc activation or inactivation could affect motor output. Future studies are necessary to characterize the time course of motor activity after CNO administration. However, all behavior tests in these studies began at the same time-point following CNO administration (30 min), and all tests were concluded within 1-h, a time frame where CNO is effective in stimulating or inhibiting neuronal activity (Urban and Roth, 2015; Gomez et al., 2017; Jendryka et al., 2019).

Future studies are necessary to determine the effects of inhibiting BLA-NAc projections in mice subjected to chronic CORT administration. We only tested the effects of Gq-DREADDs in this projection in CORT-treated mice because we hypothesized that activation would reverse the maladaptive effects of chronic CORT on reward behaviors. However, it is conceivable that inhibiting BLA CCK+ projections to NAc may actually lead to increased reward-seeking.

## Conclusions

In conclusion, here we demonstrate that BLA-NAc activation rescues deficits in reward behavior caused by chronic CORT. We also find that BLA-NAc inactivation reduced preference for sucrose. These findings are among the first to examine both reward and avoidance behaviors in BLA-NAc projection neurons, and to combine a chronic CORT paradigm with stimulation of BLA-NAc projection neurons. These results indicate that deficits in reward and motivation seen in mood disorders like MDD may be improved by activation of circuitry including BLA-NAc neurons.

## Data Availability Statement

The raw data supporting the conclusions of this article will be made available by the authors, without undue reservation.

## Ethics Statement

The animal study was reviewed and approved by Rutgers IACUC.

## Author Contributions

AD, JF, DB, and BS contributed to the experimental design, data analysis, interpretation, and wrote the manuscript. AD, JF, KS, JL, and PS performed the experiments. All authors contributed to the article and approved the submitted version.

## Conflict of Interest

The authors declare that the research was conducted in the absence of any commercial or financial relationships that could be construed as a potential conflict of interest.

## Supplementary Material

SUPPLEMENTARY FIGURE 1 **(A)** Number of active lever presses, **(B)** breakpoint ratio, and **(C)** PROG reinforcers earned (g) per gram body weight (g) are similar in a PROG session in mCherry, Gq-DREADD, and Gi-DREADD BLA-NAc mice. **(D)** Sucrose consumption (ml) per gram body weight (g) is similar in mCherry, Gq-DREADD, and Gi-DREADD BLA-NAc mice. **(E)** NSF latency was divided by home cage feeding and expressed as a ratio of latency (s) to consumption (mg). For NSF ratio, a one-way ANOVA revealed a significant effect of BLA-NAc neurons (*F*_(2,27)_ = 3.70, *p* = 0.0398). NSF ratio was increased in Gq-DREADD mice compared to mCherry control (*p* = 0.0256), while Gi-DREADD mice and mCherry control were similar (*p* = 0.6533). **(F)** Distance traveled in the OFT and **(G)** time in the center of the OFT is similar between in BLA-NAc Gi-DREADD, Gq-DREADD, and mCherry groups. Bars are mean ± SEM. **p* < 0.05.

SUPPLEMENTARY FIGURE 2 **(A)** CNO or saline pretreatment does not affect lever presses in PROG (*t*_(16)_ = 0.403, *p* = 0.6923), **(B)** PROG breakpoint ratio reached (*t*_(16)_ = 0.1853, *p* = 0.8554), or **(C)** PROG reinforcers consumed (g) per gram body weight (g; *t*_(16)_ = 0.04611, *p* = 0.9638). **(D)** CNO or saline pretreatment does not affect preference in SPT (*t*_(16)_ = 0.6722, *p* = 0.511). **(E)** For sucrose consumption in SPT (ml) compared to mouse body weight (g), consumption per gram body weight (g) was similar between CNO and saline mCherry groups (*t*_(16)_ = 0.6529, *p* = 0.5231). **(F)** CNO or saline pretreatment does not affect locomotor activity in the OFT (*t*_(16)_ = 0.4158, *p* = 0.6831), or **(G)** time in the center of the arena (*t*_(16)_ = 0.1975, *p* = 0.8459). **(H)** CNO or saline pretreatment does not affect NSF latency NSF (Log-rank Mantel-Cox test; $X_{\text{(1)}}^{\text{2}}$ = 0.390, *p* = 0.5306) or **(I)** NSF home cage feeding, where consumption per gram body weight (g) was similar (*t*_(16)_ = 0.8596, *p* = 0.4027). Bars are mean ± SEM.

SUPPLEMENTARY FIGURE 3 **(A)** Distance traveled (cm) in the open field and **(B)** time in the center of the open field in the BLA-NAc CORT cohort **(C)** NSF latency was divided by home cage feeding and expressed as a ratio of latency (s) to consumption (mg). For NSF ratio, a two-way ANOVA with CORT administration and BLA-NAc activation as between-subjects factors revealed no main effect of CORT (*F*_(1,36)_ = 3.2, *p* = 0.08), no main effect of BLA-NAc activation (*F*_(1,36)_ = 1.1, *p* = 0.296), and no interaction (*F*_(1,36)_ = 0.059, *p* = 0.809). Thus, the NSF ratio did not differ between groups, though the main effect of CORT administration showed a small trend (*p* = 0.08). **(D)** Body weights across 4 weeks of CORT administration plus three additional weeks when behavioral tests were conducted in the cohort tested in FR30, OFT, and NSF. **(E)** Volume consumed in Vehicle and CORT cages across 4 weeks of CORT administration with 3 weeks of behavioral testing in the cohort tested in FR30, OFT, and NSF. **(F)** Mean daily CORT consumed (mg/kg/day) in CORT-administered mCherry and Gq-DREADD mice. **(G)** Body weights across 4 weeks of CORT administration plus three additional weeks in the cohort tested in feeding and reward-seeking tasks. **(H)** Volume consumed in Vehicle and CORT cages across 4 weeks of CORT administration with 3 weeks of behavioral testing in the cohort tested in the feeding and reward-seeking tasks. **(I)** Mean daily CORT consumed (μg/kg/day) in CORT-administered mCherry and Gq-DREADD mice. Bars or lines are mean ± SEM. **p* < 0.05.

SUPPLEMENTARY FIGURE 4 **(A)** Representative images of BLA taken at 4× magnification from BLA-NAc cohort displaying AAV-Cre-eGFP (green), AAV-Gq-DREADD-mCherry (red), and merged overlay including DAPI (blue) to indicate nucleic acid. **(B)** Representative image of nucleus accumbens (NAc) taken at 4× magnification displaying AAV-Cre-eGFP (green) infused into the NAc. **(C)** Representative image taken at 20× magnification of BLA neurons displaying an expression of DAPI (blue), AAV-Cre-eGFP (green), and AAV-DREADD-mCherry (red). **(D)** Quantified DREADD-mCherry expression in the basolateral amygdala (BLA) in all DREADD groups. Gi-DREADD and Gq-DREADD (*n* = 10/group) from the BLA-NAc cohort. Vehicle Gq-DREADD and CORT Gq-DREADD (*n* = 10/group) from the CORT administration cohort #1. Vehicle Gq-DREADD and CORT Gq-DREADD (*n* = 10/group) from the CORT administration cohort #2. Background subtracted mean optical intensity (a.u.) taken from TIF images processed from ImageJ software from every fourth BLA section, from the approximate coordinates: −1.20 to −1.9 *y* posterior to Bregma. A one-way ANOVA between groups was insignificant (*F*_(5,54)_ = 0.3907, *p* = 0.8531), indicating expression levels were consistent across cohorts in these experiments. Bars are mean ± SD.
