# Supplementary figures and images for: Activation of Basolateral Amygdala to Nucleus Accumbens Projection Neurons Attenuates Chronic Corticosterone-Induced Behavioral Deficits in Male Mice
Source: Front Behav Neurosci. 2021 Feb 24;15:643272. doi: 10.3389/fnbeh.2021.643272 (PMC7943928; doi:10.3389/fnbeh.2021.643272)

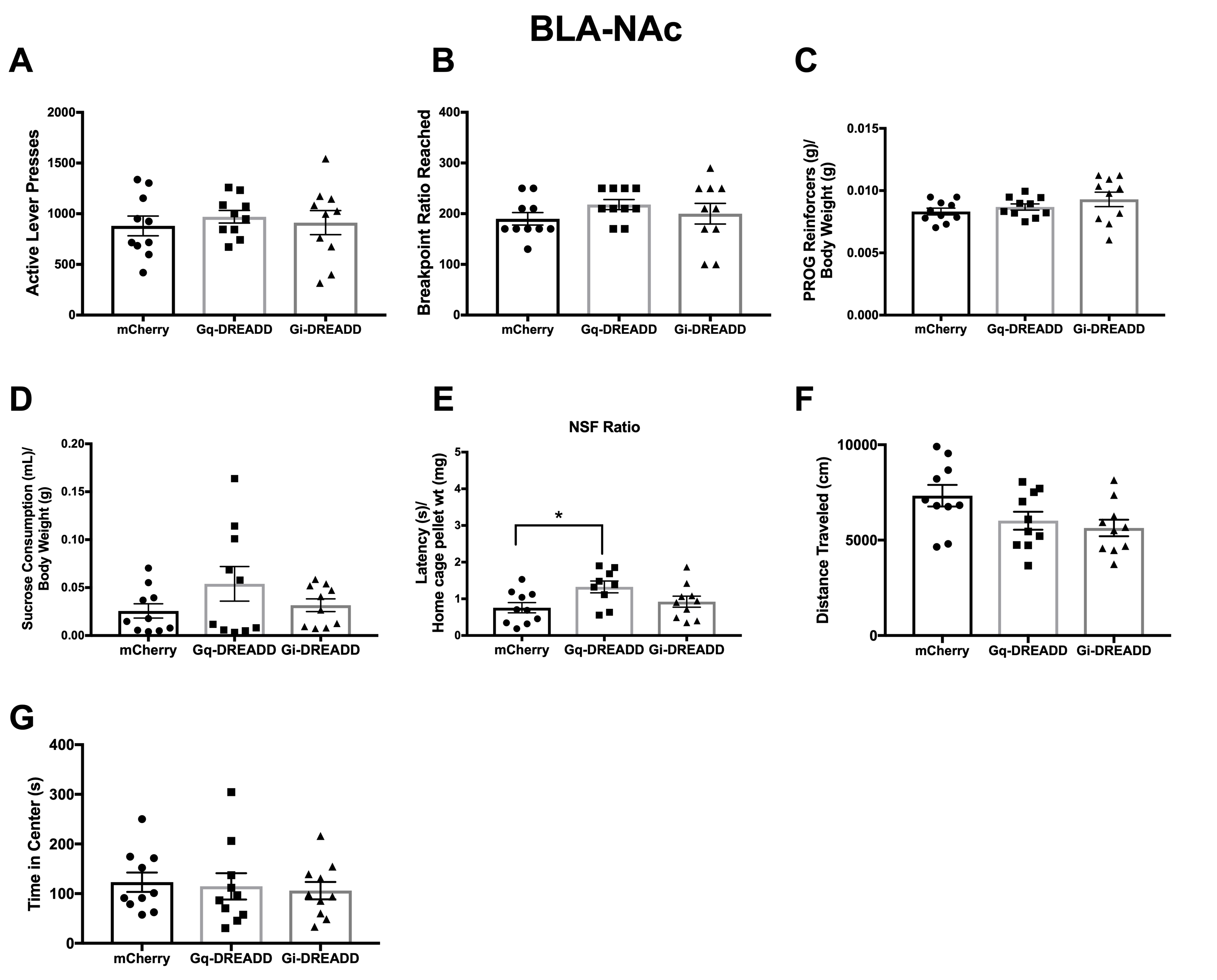

Supplement: SUPPLEMENTARY FIGURE 1 — (A) Number of active lever presses, (B) breakpoint ratio, and (C) PROG reinforcers earned (g) per gram body weight (g) are similar in a PROG session in mCherry, Gq-DREADD, and Gi-DREADD BLA-NAc mice. (D) Sucrose consumption (ml) per gram body weight (g) is similar in mCherry, Gq-DREADD, and Gi-DREADD BLA-NAc mice. (E) NSF latency was divided by home cage feeding and expressed as a ratio of latency (s) to consumption (mg). For NSF ratio, a one-way ANOVA revealed a significant effect of BLA-NAc neurons (F(2,27) = 3.70, p = 0.0398). NSF ratio was increased in Gq-DREADD mice compared to mCherry control (p = 0.0256), while Gi-DREADD mice and mCherry control were similar (p = 0.6533). (F) Distance traveled in the OFT and (G) time in the center of the OFT is similar between in BLA-NAc Gi-DREADD, Gq-DREADD, and mCherry groups. Bars are mean ± SEM. *p < 0.05. [file Image_1.TIFF]

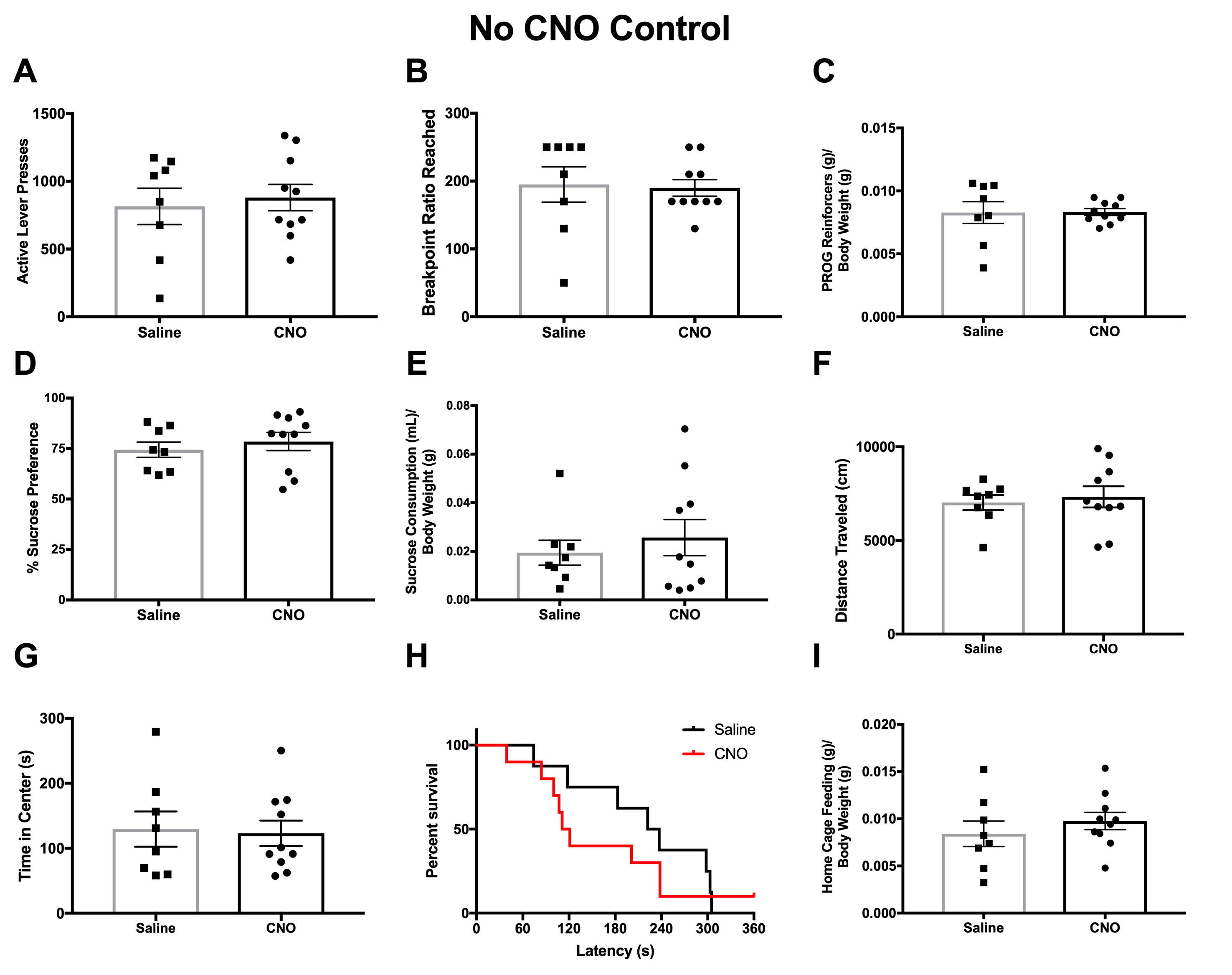

Supplement: SUPPLEMENTARY FIGURE 2 — (A) CNO or saline pretreatment does not affect lever presses in PROG (t(16) = 0.403, p = 0.6923), (B) PROG breakpoint ratio reached (t(16) = 0.1853, p = 0.8554), or (C) PROG reinforcers consumed (g) per gram body weight (g; t(16) = 0.04611, p = 0.9638). (D) CNO or saline pretreatment does not affect preference in SPT (t(16) = 0.6722, p = 0.511). (E) For sucrose consumption in SPT (ml) compared to mouse body weight (g), consumption per gram body weight (g) was similar between CNO and saline mCherry groups (t(16) = 0.6529, p = 0.5231). (F) CNO or saline pretreatment does not affect locomotor activity in the OFT (t(16) = 0.4158, p = 0.6831), or (G) time in the center of the arena (t(16) = 0.1975, p = 0.8459). (H) CNO or saline pretreatment does not affect NSF latency NSF (Log-rank Mantel-Cox test; X(1)2 = 0.390, p = 0.5306) or (I) NSF home cage feeding, where consumption per gram body weight (g) was similar (t(16) = 0.8596, p = 0.4027). Bars are mean ± SEM. [file Image_2.TIFF]

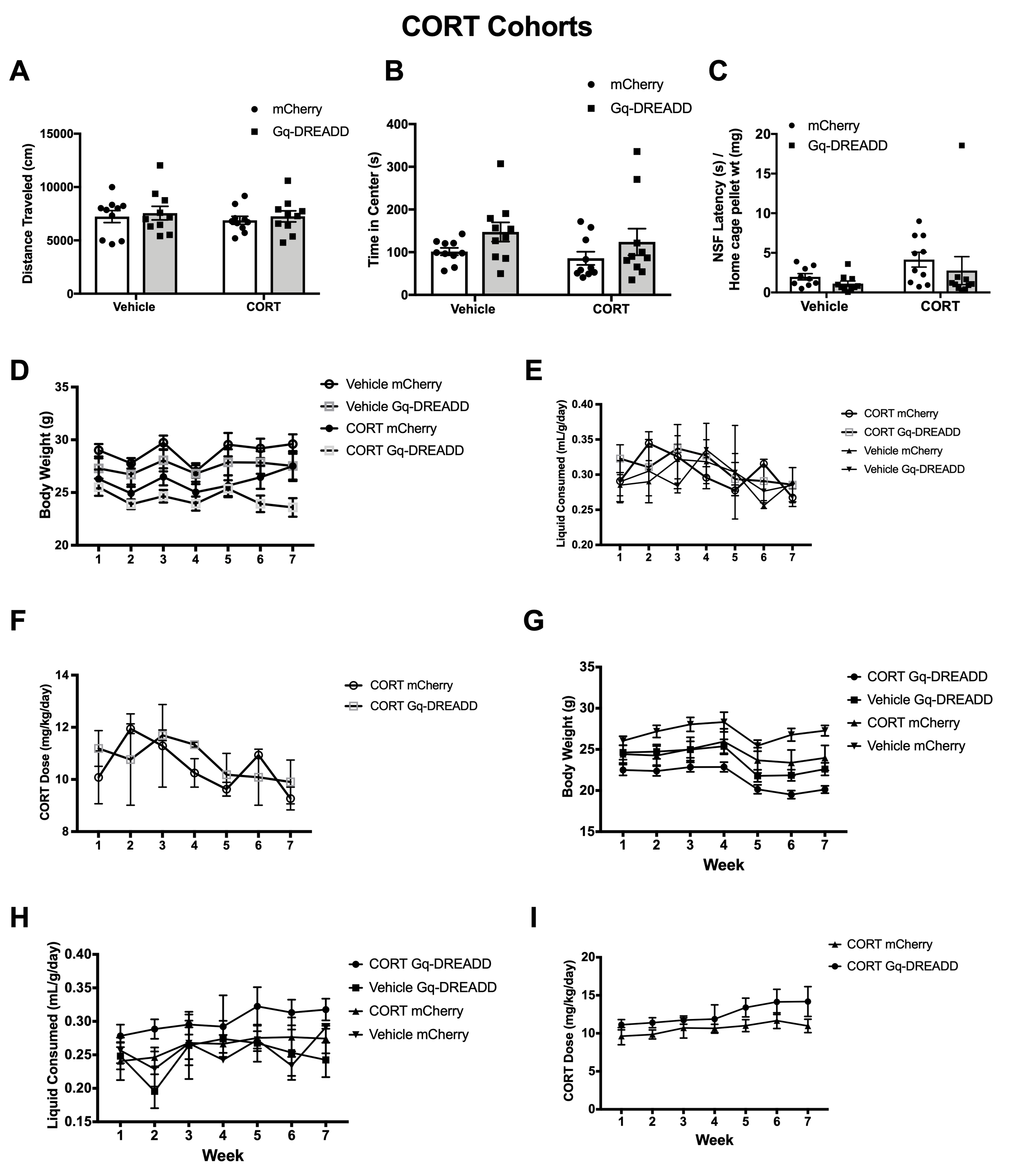

Supplement: SUPPLEMENTARY FIGURE 3 — (A) Distance traveled (cm) in the open field and (B) time in the center of the open field in the BLA-NAc CORT cohort (C) NSF latency was divided by home cage feeding and expressed as a ratio of latency (s) to consumption (mg). For NSF ratio, a two-way ANOVA with CORT administration and BLA-NAc activation as between-subjects factors revealed no main effect of CORT (F(1,36) = 3.2, p = 0.08), no main effect of BLA-NAc activation (F(1,36) = 1.1, p = 0.296), and no interaction (F(1,36) = 0.059, p = 0.809). Thus, the NSF ratio did not differ between groups, though the main effect of CORT administration showed a small trend (p = 0.08). (D) Body weights across 4 weeks of CORT administration plus three additional weeks when behavioral tests were conducted in the cohort tested in FR30, OFT, and NSF. (E) Volume consumed in Vehicle and CORT cages across 4 weeks of CORT administration with 3 weeks of behavioral testing in the cohort tested in FR30, OFT, and NSF. (F) Mean daily CORT consumed (mg/kg/day) in CORT-administered mCherry and Gq-DREADD mice. (G) Body weights across 4 weeks of CORT administration plus three additional weeks in the cohort tested in feeding and reward-seeking tasks. (H) Volume consumed in Vehicle and CORT cages across 4 weeks of CORT administration with 3 weeks of behavioral testing in the cohort tested in the feeding and reward-seeking tasks. (I) Mean daily CORT consumed (μg/kg/day) in CORT-administered mCherry and Gq-DREADD mice. Bars or lines are mean ± SEM. *p < 0.05. [file Image_3.TIFF]

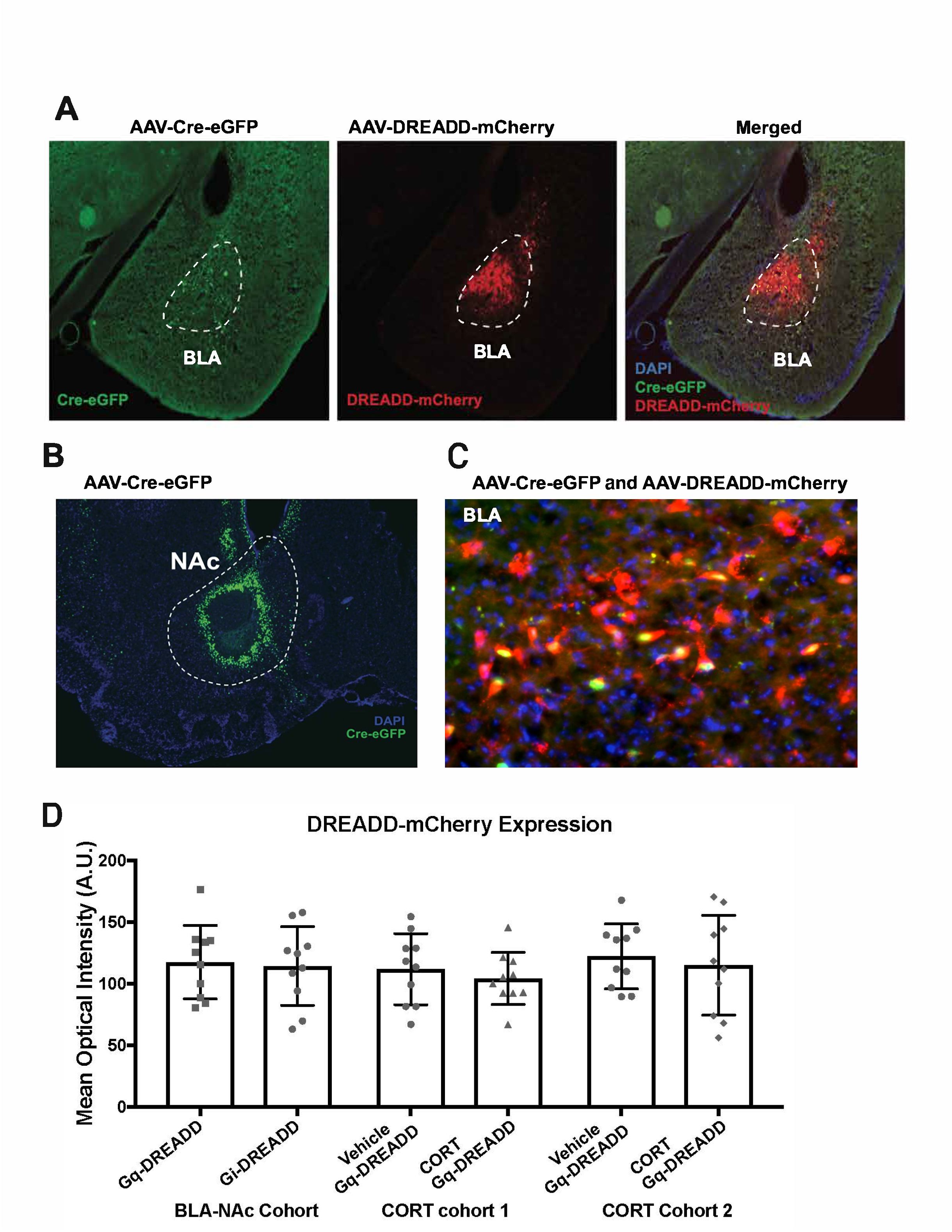

Supplement: SUPPLEMENTARY FIGURE 4 — (A) Representative images of BLA taken at 4× magnification from BLA-NAc cohort displaying AAV-Cre-eGFP (green), AAV-Gq-DREADD-mCherry (red), and merged overlay including DAPI (blue) to indicate nucleic acid. (B) Representative image of nucleus accumbens (NAc) taken at 4× magnification displaying AAV-Cre-eGFP (green) infused into the NAc. (C) Representative image taken at 20× magnification of BLA neurons displaying an expression of DAPI (blue), AAV-Cre-eGFP (green), and AAV-DREADD-mCherry (red). (D) Quantified DREADD-mCherry expression in the basolateral amygdala (BLA) in all DREADD groups. Gi-DREADD and Gq-DREADD (n = 10/group) from the BLA-NAc cohort. Vehicle Gq-DREADD and CORT Gq-DREADD (n = 10/group) from the CORT administration cohort #1. Vehicle Gq-DREADD and CORT Gq-DREADD (n = 10/group) from the CORT administration cohort #2. Background subtracted mean optical intensity (a.u.) taken from TIF images processed from ImageJ software from every fourth BLA section, from the approximate coordinates: −1.20 to −1.9 y posterior to Bregma. A one-way ANOVA between groups was insignificant (F(5,54) = 0.3907, p = 0.8531), indicating expression levels were consistent across cohorts in these experiments. Bars are mean ± SD. [file Image_4.TIFF]
